# Two-Year Results of a Five-Year Personalized Integrative Obesity Coaching Program (IBO) Based upon a Systems Health Perspective and an Evolutionary Longitudinal Study Approach

**DOI:** 10.3390/ijerph21060807

**Published:** 2024-06-20

**Authors:** Sander M. Brink, Heleen M. Wortelboer, Ard F. ten Hoff, Cornelis H. Emmelot, Tommy L. S. Visscher, Herman A. van Wietmarschen

**Affiliations:** 1 Vogellanden, Center of Rehabilitation Medicine & Special Dentistry, 8001 BB Zwolle, The Netherlands; a.ten.hoff@vogellanden.nl (A.F.t.H.); c.h.emmelot@home.nl (C.H.E.); 2 The Netherlands Organization for Applied Scientific Research (TNO), Department Work Health and Technology, 2333 BE Leiden, The Netherlands; heleen.wortelboer@tno.nl; 3 Knowledge Centre Societal Innovations, Windesheim University of Applied Sciences, 1315 RC Almere, The Netherlands; t.l.s.visscher@windesheim.nl; 4 Louis Bolk Institute, Department of Nutrition and Health, 3981 AJ Bunnik, The Netherlands; h.vanwietmarschen@louisbolk.nl

**Keywords:** obesity, complex systems approach, personalized integrative coaching program, combined lifestyle intervention, behavior change, evolutionary study design

## Abstract

This study presents the outcomes of a 5-year personalized integrative coaching program for adults with obesity (body mass index BMI ≥ 30 kg/m^2^), based upon a systems health perspective, during the first 2 years. This longitudinal study, which had an evolutionary design, included all adults who enrolled in the program. Health-related quality of life (HRQoL) was measured with the Short Form-36 (SF-36), and physical outcomes included weight, waist circumference, aerobic capacity, lipid profile, and HbA1c. Subsequently, participants completed questionnaires (e.g., the Symptom Checlist-90 (SCL-90) and the Checklist Individual Strength (CIS)). Seventy-nine adults with a mean BMI of 39.5 kg/m^2^ (SD 5.3) were included. Forty-four participants completed 2 years in the program. Compared to baseline, there were significant improvements in the SF-36 subscales ‘physical functioning’ (MD 9.9 points, 95% CI: 2.1–17.5, *p* = 0.013) and ‘general health perceptions’ (MD 9.3 points, 95% CI 2.9–15.7, *p* = 0.006). Furthermore, significant improvements in physical outcomes and psychosocial questionnaires (e.g., weight loss (MD 3.5 kg, 95% CI: 1.2–5.7, *p* = 0.003), waist circumference (MD 5.1 cm, 95% CI: 2.4–7.8, *p* < 0.001), and CIS fatigue (MD 6.8, 95% CI: 3.1–10.5, *p* = 0.001) were observed. This study highlights the importance of a systems health perspective supporting the development of a personalized integrative coaching program for adults with obesity in a ‘real-world’ setting.

## 1. Introduction

Worldwide, being overweight and obesity have become the most important public health concerns due to their association with over 200 non-communicable diseases (e.g., type 2 diabetes mellitus, cardiovascular disease, and depression), complications from COVID-19 infections, and related economic consequences [1,2]. In the Netherlands, approximately 50% of adult citizens were overweight (Body Mass Index (BMI) 25.0 to 29.9 kg/m^2^), and almost 14% of those were struggling with obesity (BMI ≥ 30.0 kg/m^2^) in 2020 [3]. It is estimated that the percentage of adults in the Netherlands who are overweight and have obesity will increase to 62% in 2040 [4].

In the Netherlands, there are a number of certified Combined Lifestyle Interventions (CLIs) designed to help people living with overweight/obesity or other risk factors to improve their lifestyle. These counseling programs provide dietary advice, physical activity training, and counseling on behavioral change over a period of 2 years [5]. The CLIs are divided into a treatment and maintenance phase and incorporate a more or less fixed mix of group and individual sessions. Although CLIs initially were promising for reducing overweight and obesity, evaluation studies have so far shown limited positive effects [6,7,8,9,10,11,12]. This is possibly due to a more or less one-size-fits-all approach with relatively low coaching frequency. Additionally, the follow-up period for CLIs is up to a maximum of 18 months, making it unclear how sustainable the results are in the long run, while it is known that weight rebound after weight loss is a significant problem [13]. Above all, lifestyle interventions often report poor attendance and high attrition rates, which limit their treatment effectiveness and overall health outcomes [14]. As a result, there is still no sustainable solution to the issue.

Obesity is a chronic complex disease [2]. Many and large inter-individual differences (e.g., coping strategies, social support, and health literacy) in physical, psychological, social, behavioral, environmental, and political factors (interacting in a non-linear manner) are the result of a long build-up due to the interaction of events, which all play a role in the development of overweight and obesity [15,16,17]. Therefore, several researchers have suggested the adoption of a systems health perspective and approach to effectively address the complexity of chronic lifestyle-related diseases such as overweight and obesity [18,19].

Systems are defined as a whole that cannot be divided into parts. The behavior of each element has an effect on the whole, and the behavior of the elements and their effects on the whole are interdependent. Obesity can be conceptualized as the outcome of a so-called multidimensional complex system of nested subsystems (e.g., individual in work, individual in a family, individual in a healthcare network) with short-term changes in patterns of behavior of both the individual and context, habits, social interrelationships, events, physiology, whereas at the same time, slow deterioration of these patterns over time can result in relatively stable adaptation strategies [16,17]. Hence, in the situation that the complex system is in a stable unhealthy state, interventions should focus on destabilizing the unhealthy structures and behaviors which underlie the unhealthy stable states, with the aim of pushing these toward healthier states [19]. For each individual with a personal pattern of organization, we expect that there are particular types of interventions that will trigger such a destabilizing effect. To deal with such dynamics and gradual differences in states, we suggested that a more personalized coaching program is needed in combination with an evolutionary study approach [18,20].

As far as we know, there is no scientific literature in which the outcomes of lifestyle coaching programs based on a system health perspective for adults with obesity have been reported. We hypothesized that an integrative personalized coaching program for adults with obesity based on a systems health perspective leads to sustainable positive changes in quality of life and physical and psychosocial outcomes. Therefore, this study aimed to explore the progress and outcomes of health-related quality of life (HRQoL) and physical and psychosocial outcomes of adults with a BMI ≥ 30 kg/m^2^ attending the first 2 years of an integrative personalized coaching program based upon a systems health perspective.

## 2. Materials and Methods

### 2.1. Study Design and Setting

A single-center, evolutionary longitudinal study started in 2015 in Vogellanden, Center of Rehabilitation Medicine and Special Dentistry (Zwolle, The Netherlands), and is still ongoing [18]. This study used the Template for Intervention Description and Replication (TIDieR) checklist and STrengthening the Reporting of OBservational studies in Epidemiology (STROBE) reporting guidelines [21,22]. The local medical ethical review board reviewed the study protocol and concluded that ethical approval within the scope of the Dutch Medical Research Involving Human Subjects Act (Central Committee on Research Involving Human Subjects is not needed (15.0224)). Written informed consent was obtained from all participants involved in the study.

### 2.2. Recruitment Method and Participants

This study reports the results of the participants enrolled between January 2015 and December 2019. General practitioners and medical specialists referred potential candidates to the rehabilitation center. Eligibility criteria were adults with obesity who had previously participated in an (indivual or group) weight loss intervention with professional support without sustainable behavioral change and/or weight loss results based upon expert opinion (stepped care principle). After referral, a rehabilitation physician and a psychologist assessed whether the candidates met the inclusion criteria.

Inclusion criteria were adults (age ≥ 18 years old) with obesity (BMI ≥ 30 kg/m^2^), including adults with non-insulin-dependent diabetes mellitus (type 2 diabetes). Because people differ in physical fitness, activities, and participation even at older ages, we decided not to set an upper age limit. Adults with current pulmonary-, cardiovascular-, internal- (including insulin-dependent diabetes mellitus), and/or psychologic (e.g., eating-) disorders that may interfere with participating in the IBO were excluded. In cases of age > 40 years old, signs of pulmonary and/or cardiovascular risk factors, or serious doubt about physical resilience, a maximum performance test was carried out prior to the start of the program. Based on the results, patients might have been excluded from the study.

A feature of the IBO is that it is continuously being developed based on the experiences of professionals, feedback of participants, and scientific knowledge. Therefore, during the first 2 years of the development of the coaching program, a maximum of 10 participants could start each year. Since 2017, between 10 to 30 participants have started participating in the IBO every year.

### 2.3. Intervention: Personalized Integrative Obesity Coaching Program (IBO)

A detailed description of the development and design of the IBO has been published previously [19]. The main characteristics of the program are described below.

The aim of the IBO is to achieve, within a timeframe of 5 years, a sustainable, healthier lifestyle for the participants. The program is characterized by its integrative, long-term, highly adaptive, and personalized coaching strategy. A combination of multiple (lifestyle) interventions and themes focusing on behavioral change, nutrition, physical activity, sleep, and stress management are offered simultaneously by a closely collaborating interdisciplinary coaching team. This team consisted of a rehabilitation physician, a psychologist, lifestyle coaches, an internal medicine physician, a sports medicine physician, a physiotherapist, a dietician, and/or a psychomotor therapist.

Participants were organized in groups of between 8 and 12 adults. In a plenary kick-off session prior to the start of the program, the participant and a representative of our center both signed a covenant in which they committed themselves to the principles of the program and intended results. During the first 3 months after the start of the program, information based on individual goals, needs, questionnaires, and physical- and laboratory measurements were put together and used to develop a personal (coaching) strategy for each participant. Therefore, the content and coaching frequency could vary among participants. Changes toward a healthier lifestyle of the participant were achieved by setting small goals and, when these goals were achieved, setting new small goals. During the first 2 years of the 5-year IBO, the backbone consisted of group coaching sessions (12 to 24) that took place monthly. These sessions lasted for two hours and were facilitated by a psychologist and one of the lifestyle coaches. A comprehensive manual was used to address the aforementioned themes and other relevant (lifestyle) topics, and experiences between participants were shared. Participants received a homework assignment prior to each group coaching session, such as reading specific content or preparing a presentation on different types of eating behaviors.

Additionally, each participant was offered individual coaching sessions supervised by a psychologist, dietician, and/or lifestyle coach. These 1-hour sessions (two to four sessions per month) were tailored to the personal goals and needs of the participant. If mental issues were identified during the intake session at least two sessions with a psychologist are scheduled. Furthermore, an individual intake session with a dietitian is conducted to identify any particularities in nutrition behavior and to tailor the guidance accordingly. Nutritional advice is not aimed at following a diet but at being able to sustainably maintain the consumption of healthier food products and eat smaller portions at fixed times during the day. In addition, participants learned strategies to avoid (reduce) purchasing unhealthy (ultra-processed) food products in, for example, supermarkets.

To increase participants’ physical activity, onsite 1-hour sports sessions were held at least twice a week. In groups, participants could experience various exercise and sports activities. The aim of these sports sessions was to find a sport or exercise activity that the participants were willing and able to practice in their own environment (e.g., weekly walks with other participants of the IBO).

It is clear that social support has a positive influence on maintaining healthy behavior [23,24]. Therefore, partners, family members, or friends were invited to participate in, for example, cooking workshops and sports activities. By involving them in the program, it should become easier for the participants to apply what they learned at home.

Most individual and group sessions were scheduled in the first year of the program. The participants then applied what they learned, and the support from a lifestyle coach was phased out. If necessary, participants could contact their lifestyle coach up to 5 years after the start.

The progress of each participant was evaluated and discussed every 6–8 weeks with the core team. In between these evaluations, coaches were allowed to fine-tune their approach. As long as participants participated in the program, progress was monitored at pre-arranged times. Depending on these evaluations and the measurements (including questionnaires), the treatment strategy will be determined. For example, for one participant, the emphasis was on the support of the psychologist, while for another participant, improving physical activity was the most important part of the program.

### 2.4. Monitoring Plan

At baseline, sociodemographic characteristics were recorded. Validated questionnaires and physical outcome measures were used to determine changes over time. The primary outcome measure was HRQoL assessed with the Short-Form Health Survey (SF-36: 0–100) [25]. This questionnaire consists of an item on health transition and eight multi-item subscales for physical function (10 items), social function (2 items), role limitations due to physical health problems (4 items), role limitations due to emotional problems (3 items), mental health (5 items), vitality (4 items), pain (2 items), and general health (5 items). The scores for the eight subscales were calculated based on the responses to the items on each scale using standard SF-36 scoring algorithms [26]. The summary scores range from 0 to 100, where higher scores indicate better quality of life. In addition, the eight subscales can be aggregated into two component summary scales: the Physical Component Summary (PCS) score to assess physical health and the Mental Component Summary (MCS) score to assess mental health [26]. In this study, using standard SF-36 scoring algorithms, the scores for all eight subscales were calculated based on the responses to the items on each scale [25]. The scores for each subscale were then standardized using a z-score transformation [26]. Furthermore, the SF-36 subscale means and standard deviations for Dutch adult population norms were used [27]. These standardized scores of the eight subscales were aggregated to calculate the PCS and MCS scores. A three-to-five-point increase in PCS or MCS score is considered clinically important [28].

Secondary outcomes were weight (kg), BMI (kg/m^2^), and body and visceral fat (%), the latter measured with a four-point bioelectrical impedance device of Tanita RD-545 (Tanita, Tokyo, Japan). Waist and hip circumferences (cm) were measured with a measuring tape [29]. Maximal aerobic capacity (VO_2_-max) was predicted based on the submaximal cycle ergometer aerobic fitness test according to the protocol of Åstrand (mL/kg/min) [30]. All physical outcomes were measured by the lifestyle coaches delivering the intervention, who were trained to perform the measurements in a standardized way. Automated enzymatic colorimetric method was used to determine total cholesterol-(TC), high-density lipoprotein-(HDL-c), low-density lipoprotein-(LDL-c), and triglycerides-(TG) levels, which were recorded in mmol/L. In addition, a cholesterol ratio (TC/HDL-c) was calculated. The high-pressure liquid chromatography (HPLC) method was used to determine median hemoglobin A1c (HbA1c) and registered in mmol/mol. The cholesterol ratio and HbA1c were compared with local reference values (<5.0 and between 20–42 mmol/mol, respectively). In addition, participants completed the Rosenberg Self-Esteem Scale (RSES) [31], the Checklist Individual Strength (CIS) [32], the Utrecht Coping List (UCL) [33], and the Symptom Checklist (SCL-90) [34]. As long as participants were participating, the outcomes were collected seven times during the first 2 years (at baseline and at 3-, 6-, and 9 months, and after one, 1.5- and 2 years after baseline). The primary endpoint was 2 years after baseline.

### 2.5. Data Management and Statistical Analyses

A custom-designed case report form was built in a web-based clinical database (Research Manager, Deventer, The Netherlands) and was used for sending questionnaires by e-mail and data storage. The Statistical Package for Social Sciences, version 24.0 (IBM SPSS Inc., Chicago, IL, USA) was used for statistical analyses. Continuous variables are presented as means with standard deviation (SD). Dichotomous and categorical data are described as frequencies with percentages. Characteristics of the participants and outcome measures are presented as frequencies (percentage) and means (standard deviation). Baseline and 1 year and baseline and 2 years were compared using paired sample *t*-tests. Thereafter, effect sizes (ES) for correlated measurements between baseline and the 1 year, and between baseline and the 2 years measurements were calculated ((mean after the one and 2 year(s) minus the mean at baseline) divided by (the standard deviation of the difference, respectively)) [35]. The effect sizes were categorized as follows: very small (0.01), small (0.20), medium (0.50), large 0.80), very large (1.20), and huge (2.00) [36].

The percentages of participants who succeeded in achieving a weight reduction of at least 5% after 1 and 2 year(s) were calculated. In addition, percentages were calculated for those who had at baseline TC/HDL-c ≥ 5.0, HbA1c levels ≥ 42.0 mmol/mol, RSES- ≤ 15, and CIS fatigue ≥ 27 scores and succeeded to reach normal levels or scores at 2 years. Chi^2^ tests were used to compare categorical data.

For those participants who discontinued the program within 2 years, differences between baseline and the last measurement of the SF-36 (PCS and MCS), weight, BMI, TC/HDL-c, and HbA1c levels were calculated and tested for differences with paired sample *t*-tests. The group that completed 2 years in the program was compared with the group that discontinued the program at baseline with independent *t*- and Chi^2^-tests.

Per-protocol analyses were performed. A two-sided *p*-value < 0.05 was considered statistically significant.

## 3. Results

**Figure 1:**
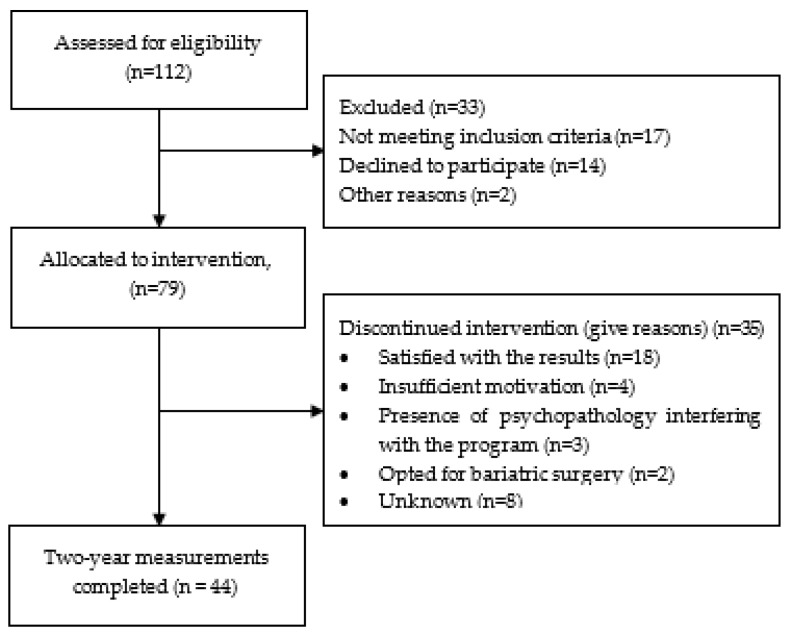
Flowchart of the study.

Until December 2019, 112 potential candidates were assessed for eligibility. Seventy-nine adults divided over eight groups were enrolled in the program. The mean age was 46.3 years old (SD 12.6), the mean BMI was 39.5 kg/m^2^ (SD 5.3), and 61 of the adults were women (Table 1). Attendance rates after half, 1, 1.5, and 2 year(s) were 96.2% (*n* = 76), 78.5% (*n* = 62), 65.8% (*n* = 52), and 55.7% (*n* = 44), respectively. In consultation with the coaching team, 29 females (48%) and 6 males (33%) decided to discontinue the program within the first 2 years (mean 11.9 months (SD 5.0)). The mean age of these study dropouts was 44.4 years old (SD 12.5), and the mean BMI was 40.9 kg/m^2^ (SD 9.7) at baseline. The BMI of the dropouts was significantly higher compared to those who continued participating (MD 2.4 kg/m^2^, 95% CI: 0.1–4.7, *p* = 0.041). The main reason mentioned for discontinuing the IBO was that participants were satisfied with the outcomes at that moment and/or felt adequately informed to maintain a healthy lifestyle. Other reasons were that the program was too intensive or too time-consuming, and/or participants were dissatisfied with the achieved weight loss and opted for bariatric surgery. A flowchart of the study is presented in Figure 1.

### 3.1. Primary Outcome: Health-Related Quality of Life

An overview of the HRQoL scores during the first 2 years is presented in Table 2. No significant differences of the PCS and MCS between baseline and 1 year, and between baseline and 2 years, were observed. Additional analyses showed that the subscales physical function (MD 11.5, 95% CI: 6.2–16.9, *p* < 0.001), social function (MD 7.3, 95% CI: 1.2–13.4, *p* = 0.020), general mental health (MD 5.5, 95% CI: 1.1–9.8, *p* = 0.016), vitality (MD 8.4, 95% CI: 3.2–13.5, *p* = 0.002), general health perceptions (MD 11.6, 95% CI: 7.0–16.2, *p* < 0.001), and the item health transition (MD 28.0, 95% CI: 17.9–38.0, *p* < 0.001) improved significantly after 1 year. Furthermore, compared to baseline, significant improvements in the subscales physical functioning (MD 9.9, 95% CI: 2.1–17.5, *p* = 0.013) and general health perceptions (MD 9.3, 95% CI: 2.9–15.7, *p* = 0.006) were observed after 2 years. For those who discontinued the program, no significant differences of the PCS and MCS between baseline and the last measurements were observed. Analysis showed significant improvements in the subscales physical function (MD 8.3, 95% CI: 1.4–15.2, *p* = 0.019), general health perceptions (MD 9.9, 95% CI: 3.0–16.7, *p* = 0.006), and the item health transition (MD 22.9, 95% CI: 13.4–32.5, *p* < 0.001).

### 3.2. Secondary Outcomes

#### 3.2.1. Physical Outcomes

An overview of the physical outcomes and aerobic capacity during the first 2 years is presented in Table 3. Data indicate that after 1 year compared to baseline, participants showed significant improvement in weight (MD 5.7 kg, 95% CI: 3.5–8.0, *p* < 0.001), BMI (MD 1.9 kg/m^2^, 95% CI: 1.2–2.7, *p* < 0.001), waist circumference (MD 6.8 cm (6%), 95% CI: 4.5–9.0, *p* < 0.001), hip circumference (MD 5.4 cm, 95% CI: 3.4–7.4, *p* < 0.001), body fat (MD 3.2%, 95% CI: 2.0–4.4, *p* < 0.001), visceral fat (1.6%, 95% CI:0.7–2.4, *p* < 0.001), and VO_2_-max (2.4 mL/kg/min, 95% CI: 0.4–4.3, *p* = 0.018). Furthermore, after 2 years compared to baseline, a significant decrease in weight (MD 3.5 kg, 95% CI: 1.2–5.7, *p* = 0.003), waist circumference (MD 5.1 cm (4.3%), 95% CI: 2.4–7.8, *p* < 0.001), hip circumference (MD 5.9 cm, 95% CI: 4.2–7.6, *p* < 0.001), and body fat (MD 2.0, 95% CI: 0.7–3.4, *p* = 0.004) were observed. In addition, participants showed a significant improvement in maximal aerobic capacity after 2 years (MD 4.0 mL/kg/min, 95% CI 2.1–5.9, *p* = 0.030). Overall, after the first 2 years of the program, 30 participants (68.8%) lost weight. A total of 16 of them lost weight between 0 and 5% (MD 2.7%, 95% CI: 0.7–5.0), and 14 participants had a weight reduction of 5% or more (MD −10.1%, 95% CI: 5.1–18.2) compared to baseline. Participants who discontinued the program showed a significant weight reduction between the baseline and the last measurement (MD 2.8 kg, 95% CI: 0.1–5.6, *p* = 0.041). In accordance with weight reduction, the BMI between baseline and the last measurement decreased significantly (MD 0.9, 95% CI: 0.0–1.9, *p* = 0.048).

#### 3.2.2. Lipid Profile and HbA1c

An overview of TC, HDL-c, and HbA1c during the first 2 years is presented in Table 4. Data indicate that no significant differences in the lipid profile and HbA1c were observed over time. In 19 participants, a cholesterol ratio equal or above 4.0 was observed at baseline. In 14 participants, a HbA1c of at least 42.0 mmol/mol was observed at baseline. With regard to cholesterol ratio, 8 out of 19 of the participants with a too high value before the start of the program changed to a desired level below 5.0. In addition, HbA1c levels of 5 out of 14 participants with a too high level at the start decreased to a value of 42.0 mmol/mol or less. At the start of the program, nine participants were taking medicine for type 2 diabetes. One of them was able to stop the use of metformin during the first year after the start of the program.

For those who left the program, no significant differences between the baseline and last measurements were found for cholesterol ratio (MD 0.1, 95% CI: −0.2–0.4, *p* = 0.512) and HbA1c (MD −0.2, 95% CI: −2.3–1.9, *p* = 0.838). In 4 out of 11 participants, the cholesterol ratio improved to a level below 5.0. In addition, out of six participants’ HbA1c improved to a level below 42.0 mmol/mol.

#### 3.2.3. Psychosocial Outcomes

An overview of the RSES, CIS, SCL-90, and UCL scores during the first 2 years is presented in Table 5. Between baseline and 1 year, significant improvements in the total CIS-sore (MD 12.0, 95% CI: 5.1–18.8, *p* < 0.001), fatigue subscale score of the CIS (MD 8.6, 95% CI: 5.1–12.2, *p* < 0.001), and RSES (MD 1.6, 95% CI: 0.4–2.7, *p* = 0.008) were observed. In addition, significant improvements after 1 year of the following subscales of the SCL-90 anxiety (MD 1.1 points, 95% CI: 0.367–1.911, *p* = 0.005), depression (MD 4.3, 95% CI: 2.1–6.4, *p* < 0.001), somatization (MD 2.1, 95% CI: 0.4–3.7, *p* = 0.015), and interpersonal sensitivity (MD 4.7, 95% CI: 1.9–7.4, *p* = 0.002) and UCL avoidance (MD 1.5, 95% CI: 0.7–2.9, *p* = 0.001) and passive reaction pattern (MD 1.1, 95% CI: 0.4–1.7, *p* = 0.002) were observed. Between baseline and 2 years, significant improvements in the subscale fatigue (MD 6.8, 95% CI: 3.1–10.5, *p* = 0.001) of the CIS and the subscale avoidance (MD 1.4, 95% CI: 0.4–2.5, *p* = 0.011) of the UCL were observed.

However, a significant decrease in the number of participants with a RSES score below 15 points at baseline (22%) to 2 years (15%) was observed (Chi^2^ = 6.619, *p* = 0.034). In addition, a significant reduction of the subscale fatigue of the CIS questionnaire between baseline and one 1 (MD 8.6, 95% CI: 5.1–12.2, *p* < 0.001) and between baseline and 2 years (MD −6.8, 95% CI: −10.5–−3.1, *p* < 0.001) were observed. In addition, a significant reduction of 81.1% at baseline to 68.6% after 2 years of the participants with a fatigue score below 27 was observed (Chi^2^ = 4.172, *p* < 0.041).

## 4. Discussion

The objective of this study was to explore the results over a period of 2 years of adults with obesity participating in the IBO, a 5-year personalized integrative coaching program designed from a systems health perspective. From this perspective, the transition from an overweight toward a more healthy state needs to be accompanied by multiple changes in psychosocial and physical variables depending on the individual. Therefore, our hypothesis is not solely based on a fast reduction of weight. Instead, we built our program upon a biopsychosocial causal loop model in which a variety and multiple determinants of obesity were represented, as well as interactions between these determinants [18]. A transition journey can be very personal and is characterized by sudden changes in behavior (critical transitions). Therefore, in the IBO, each individual was asked to set personal goals, which are often not only weight loss or physical improvement. We found significant improvements regarding quality of life (subscales ‘physical function’, ‘social function’, ‘mental health’, ‘general health’, and ‘health transition’), all physical outcomes (e.g., weight and waist circumference), self-esteem and fatigue during the first year of the IBO. Although the outcomes then deteriorated again, quality of life (subscales ‘physical function’ and ‘general health’), and physical outcomes (weight, BMI, waist-/hip circumference, body fat, and VO_2_-max) remained significantly improved after 2 years compared to baseline. In addition, a substantial portion of the participants with abnormal lipid profiles and/or HbA1c levels at baseline showed improvement during the first 2 years.

The IBO is characterized by an evolutionary design and the option to receive personalized coaching toward a healthier lifestyle for up to 5 years. Additionally, the focus is on the participant’s needs, progression, and behavioral changes rather than solely on the intervention itself. To the best of our knowledge, there is currently no comparable 5-year CLI coaching program offered within rehabilitation centers. Two-year CLIs for adults with overweight/obesity with or without diabetes are provided in primary healthcare settings in the Netherlands. Despite differences in, for example, setting, target populations, and design, it is interesting to compare the 2-year results of the IBO with current Dutch CLIs.

The primary outcome of the IBO is an improvement in quality of life. It is known that obesity is associated with lower quality of life [37]. The mean scores of the SF-36 subscales of the IBO participants at baseline ranged from 47.2 to 72.1 points, which are much lower compared to Dutch norm scores, which ranged from 69.6 to 93.9 [38]. This reflects the extent to which the quality of life in the studied population is affected. After 24 months, small effect sizes of the PCS (0.16) and MCS (−0.16) and significant improvements were observed on the subscales ‘physical function’ (9.6 points) and ‘general health’ (8.0 points). Remarkably, the participants of the IBO improved the most on ‘health transition’. This subscale increased significantly after the first 12 months with 27.8 points and then remained stable. Although beneficial improvements in quality of life were observed, many were non-significant after 2 years. Participants of the SLIMMER intervention improved by 2.7 points and 7.0 points on the ‘physical function-’ and ‘general health’ subscales of the SF-36 after 1.5 years compared to baseline [11]. Although ‘health transition’ during the first year of SLIMMER did not improve as strongly as in the IBO, the participants of SLIMMER improved significantly (15.8 points) and remained stable for up to 1.5 years [37]. Hence, it might have been more appropriate to use a disease-specific questionnaire instead of the SF-36 questionnaire. Generic instruments like the SF-36 are less sensitive to changes in HRQoL [38]. An alternative questionnaire could be the Obesi-Q, which can be used in research and clinical practice to assess weight loss treatments from a patient with an obesity perspective [39]. This questionnaire is currently (2023) recommended in the latest Dutch guideline, ‘Overweight and Obesity in Adults and Children’ [40]. While small effect sizes on the PCS and MCS scores (SF-36) after 2 years were observed in the IBO study, the Cool program demonstrated an overall medium improvement (ES 0.32) in quality of life as measured with the EQ5D [10].

One and two year(s) after baseline, participants of the IBO achieved a mean weight loss of 7.4 kg (6%) and 3.5 kg (3%), respectively. These findings are favorable compared to what was observed in previous studies of the SSiB-, CooL-, SLIMMER- and BeweegKuur-interventions, in which body weight reduction varied between 1.8 kg and 3.0 kg (tussen 0.9–18 maanden) [8,10,11,12]. However, the observed weight reduction was lower compared to Reverse Diabetes2–CLI–Online-, X-fittt-, and Reverse Diabetes2-interventions, where participants lost on average between 5.4 kg and 10.6 kg [6,7,9]. A previous study showed that people need to lose more than 20% of their weight to achieve minimal clinically important changes of five points on the PCS and MCS scores of the SF-36 in HRQoL [29]. These limited weight reductions reported in those studies may explain the minor changes in quality of life after 2 years. Nevertheless, it is still relevant, as several studies have shown that even a limited reduction in weight leads to health benefits [41,42,43,44,45].

A reduction in waist circumference of 5% or more is considered clinically relevant in the short term, and maintaining a reduction in waist circumference of ≥3% from baseline is considered clinically relevant in the long term [46]. The IBO results in statistically significant and clinically relevant reductions in waist circumference at 1 and 2 year(s) after baseline, with reductions of 6.9 cm (6.0%) after 1 year and 5.2 cm (4.3%) after 2 years.

The reduction in waist circumference that was achieved in other comprehensive lifestyle interventions is larger compared to the IBO and ranged between 3.5 cm and 9.7 cm at the end of the intervention [6,7,8,9,11,12].

The mean BMI of participants in the IBO was higher compared to other Dutch CLIs. Additionally, individuals were eligible for enrollment in the IBO only if they had previously participated in a weight loss intervention with professional support without lasting results (such as sustainable weight reduction or the adoption of a healthy lifestyle). This criterion led to the inclusion of participants in the IBO with a higher BMI (mean 39.5) compared to the mean baseline BMI (ranging from 30.2 to 36.2 kg/m^2^) in other CLIs [8,9,10,13]. In this context, the personalized approach of the IBO for this group of patients seems successful. The IBO is not a CLI but rather a more personalized, evolutionary guidance with a strong focus on the client’s needs and context. It is supported by a diverse coaching team and monitored using a set of outcome measures. Through a multi-year approach, the aim is also to investigate whether, from this longitudinal data, client’s own choices for specific actions/coaching, subgroups, and ‘patterns’ can be detected that support improvement and a more personalized approach for subgroups (which can only become apparent from the data and patterns). With this, we attempt to approach the essence of a (‘self-organizing’) systemic approach, in which the lifestyle coach does not dictate what the client should do (an ‘intervention’ in the classical sense of the word) but rather, a coaching team guides the client (and their environment, including the coaching team, peers, family, and social relationships) to collaboratively make the right choices and timely adjustments for maximum positive effectiveness.

Frequently measuring quality of life, physical outcomes (including HbA1c and lipid profile), and psychosocial outcomes can support personalizing the program. We could not find studies of any other lifestyle intervention based upon a system health perspective in which the same questionnaires were administered. Nevertheless, we expect that these results are important and can provide more insight into the interactions between physical, mental, social, and environmental determinants of health.

**Adaptations to the Initial Design of the Coaching Program**

The adaptive nature of the IBO allowed us, based on conversations with participants and professionals, to improve the program continuously. This fits with a study design based upon a systems health perspective, in which collective growth, resilience, and adaptation play an important role. A systems health perspective includes letting go of control and letting behavior within social relationships emerge by designing conditions and infrastructures that promote emergence. It also shifts the perspective from the individual to social relationships [47]. Compared to the initial design, the most important changes of the program were related to the conditions for participating, the monitoring plan, and the organization (including being able to continue offering the program during the COVID-19 pandemic). In order to include a more homogeneous group with as little interfering comorbidity as possible, we decided to lower the allowed maximum BMI to 45 kg/m^2^ from group 5 and beyond. In contrast to groups 1 to 5, all participants from groups 6 and beyond underwent a maximum performance test by a sports medicine physician. These test data were used to develop personalized training programs to improve training effectiveness.

Several changes to the monitor plan have been made with regard to food diaries, wearables, and questionnaires. In groups 1 to 4, we noticed that data gathered with food diaries were incomplete or unreliable. Discussions with participants showed that they already had to register food diaries many times in the past, which has not led to sustainable weight loss and caused stress. Therefore, we decided to only use the diaries when the lifestyle coach thought it was of added value in consultation with each individual participant. We also inform participants that self-monitoring of nutritional intake for at least 3 days per week may be beneficial for supporting long-term maintenance [48]. Subsequently, the participants are sufficiently informed to decide whether or not to monitor their nutritional intake.

There were doubts about the accuracy of the used wearables (e.g., the recorded minutes that participants were active during the past week were improbably low or high in several cases). Therefore, we decided that the wearables no longer needed to be worn by all participants. This decision was supported by scientific literature showing that the added value of wearables for most people with obesity is not proven [49,50]. Therefore, a wearable was only worn by participants who indicated that the wearable helped them to stimulate exercise behavior. In addition, the IBO is distinguished because not only professionals encourage participants, but participants also encourage each other. The combination of the high frequency of coaching-/sports sessions and peer support motivates participants to increase their physical activity. Finally, we noted that, for some participants, it was difficult to complete the questionnaires at the planned frequency. For that reason, although the questionnaires remained the same, we reduced the frequency of questionnaires that participants had to complete each measurement. Since 2016, participants have completed the RSES, CIS, and UCL three (baseline, 6 months, and 1 year), instead of five times, during the first year of participating in the program.

In March 2020, the smartphone (iOS and Android) ‘Vogellanden Vitaal’ app (version 1.0) was implemented. This app brought all elements of the program, such as recipes, (relaxation) exercises, and workshops together. In addition, participants obtained insight into their personal changes (e.g., HRQoL, weight, and lipid profile/HbA1c). A lifestyle community from all participants has been added to the app in which they were able to communicate with other participants within their own group.

Participants in groups 5 to 8 started participating within 2 years before the start of the COVID-19 pandemic. For this reason, the program was offered differently than planned in 2020 and partly in 2021. Individual- and group sessions were initially mainly offered digitally (e.g., telerehabilitation). A study has shown that offering telerehabilitation could be an effective, safe, and viable alternative in adults with overweight and obesity [51]. Since January 2021, the sessions mainly took place outside, where participants could keep a sufficient distance from each other.

To further improve the IBO, Acceptance and Commitment Therapy (ACT) will be added. ACT is a form of behavioral therapy that can be used as a coaching tool to help participants manage anxieties, negative emotions, and feelings [52]. ACT teaches individuals to accept obstacles in life rather than avoid them (acceptance). The positive effects of this acceptance include having more energy and space for the things that matter (commitment).

**Strengths and Limitations**

Strengths of the study include its ‘real-world view and setting’ for adults with obesity who have previously not been successful in changing their lifestyle. In addition, the utilization of a comprehensive set of outcome measures and frequent longitudinal assessments offers us a holistic perspective on participants’ overall health status across the six key domains (mental, emotional, physical, metabolic, nutritional, and social) of the systems health model of obesity [19]. This approach allows us to collect the data needed to identify the most optimal strategy based on identified patterns and effective client routes.

Some limitations also have to be mentioned. Including a control group in the design would have been beneficial in terms of making strong statements regarding the effectiveness of the IBO. However, the primary focus of the IBO was to co-create and evaluate an interdisciplinary evolutionary 5-year lifestyle intervention. It seemed unethical to the authors to assign highly motivated adults with obesity to a control group.

Furthermore, the proportion of participants who dropped out of the IBO because they were dissatisfied with the outcome was 18% and 22% at 9 months and 2 years, respectively. In contrast, the expectation was that a personalized lifestyle intervention tailored to the participants’ needs would result in low dropout rates. However, dropout rates in the IBO were high compared to current CLIs in which dropout rates ranged from 1% to 13% within the first 10 months of the intervention (treatment phase) [6,9,10]. The reason for the high dropout rate could be that participants who discontinued IBO had a significantly higher BMI at baseline. This finding is consistent with previous studies where a high BMI has been reported as one of the most prominent predictors of adherence or success [14,53]. Nevertheless, the IBO participants had previously not been successful after participating in another (lifestyle) intervention. From this point of view, the dropout rate from this program can be considered low.

To enhance the reliability of the study’s findings, it is recommended to supplement self-report measures with objective measures or additional validation methods. Objective measures, such as biomarkers or physical assessments, could provide more accurate and unbiased data regarding participants’ outcomes, including quality of life and eating behavior. Furthermore, developing and implementing reliable tools or methods to collect comprehensive and accurate data on participants’ dietary habits would significantly enhance the program’s understanding of the relationship between nutrition, lifestyle changes, and overall outcomes. Additionally, analyzing the personal goals of the participants and their achievements using tools such as the Goal Attainment Scaling will provide more insight into the results of a personalized program [54].

From an analytical perspective, the non-random nature of missing data (MNAR) in this per-protocol analysis may introduce attrition bias. The participants who were able to stick with the program were probably stronger in terms of psychological aspects, such as perseverance, than those who did not participate or dropped out. Therefore, it may be that the positive changes before and after the project were also due to these psychological aspects of perseverance. Moreover, the study’s generalizability is limited by the relatively small number of included participants. However, we are currently scaling up the IBO with the aim of including up to 40 participants annually.

**Future Study**

This explorative evaluation of the results focused on the outcomes at the group level. In future studies, it would be intriguing to discover outcomes at the individual level, which could provide more insight into the interactions between physical, mental, social, and environmental determinants of health and inter-individual changes over time. Additionally, after a longer period with more participants, we will reanalyze the data.

## 5. Conclusions

This explorative study demonstrates that the IBO, a 5-year personalized integrative obesity coaching program based upon a systems health perspective and an Evolutionary Longitudinal Study Approach, is successful during the first 2 years in changing aspects of QoL and physical health-related outcomes for participants who have previously not been successful. In addition to improved health-related quality of life (physical function and general health), a significant weight reduction and significant improvements in weight-related outcomes (such as waist circumference and body fat) were also achieved. Furthermore, this study shows that a systems health perspective can support the development of a personal lifestyle program in a ‘real-world’ setting, providing more insight into the interactions between physical, mental, social, and environmental determinants of health.

## Figures and Tables

**Table 1:** Baseline characteristics of the allocated participants and the group who completed 2 years compared to the group who discontinued the IBO at baseline (*n* = 79).

	Allocated Study Population (*n* = 79)	Completed (*n* = 44)	Discontinued (*n* = 35)	*p*-Value
Age (years), mean (SD)	46.3 (12.6)	47.8 (12.7)	44.4 (12.5)	0.238
Sex, *n* (%)				0.286 ^†^
Male	18 (23)	12 (27)	6 (17)	
Female	61 (77)	32 (73)	29 (83)	
Short-form 36 (0–100)				
PCS score	42.6 (9.7)	43.5 (10.7)	41.5 (9.0)	0.390 ^#^
MCS score	47.4 (11.2)	45.6 (12.1)	49.8 (9.7)	0.103 ^#^
Weight (kg), mean (SD)	117.3 (18.3)	114.0 (16.6)	121.4 (19.7)	0.073 ^#^
BMI (kg/m^2^), mean (SD)	39.5 (5.2)	38.5 (4.7)	40.9 (5.6)	0.041 ^#^
BMI grade, *n* (%)				0.279 ^†^
Grade 1 (30–34.9)	17 (22)	11 (25)	6 (17)	
Grade 2 (35–39.9)	27 (34)	17 (39)	10 (29)	
Grade 3 (≥40)	35 (44)	16 (36)	19 (54)	
Type 2 diabetes, *n* (%)	9 (11)	4 (44)	5 (56)	0.521 ^†^
Education, *n* (%)				0.665 ^†^
Lower	9 (11)	6 (14)	3 (9)	
Intermediate	39 (49)	20 (46)	19 (54)	
High	31 (39)	18 (41)	13 (37)	
Caucasion ethnicity, *n* (%)	77 (97)	43 (98)	34 (97)	0.870 ^†^

Abbreviations: PCS-score, Physical Component Summary score; MCS: Mental Component Summary score. ^#^ Unpaired *t*-test, ^†^ Chi^2^-test between the group who completed 2 years compared to the group who discontinued the IBO at baseline.

**Table 2:** Primary outcome: Physical- and Mental Component Summary scores, eight subscale scores and the item health transition of Health-related Quality of Life (SF-36) during the first 2 years of the IBO (*n* = 44).

	Baseline	3 Months	6 Months	9 Months	1 Year	*p ^¥^*	ES	1.5 Years	2 Years	*p ^¥^*	ES
PCS score	43.7 (10.7)	47.2 (9.7)	48.9 (9.4)	48.9 (6.9)	47.8 (9.3)	0.166	0.29	46.6 (9.4)	47.2 (9.6)	0.214	0.16
MCS score	45.6 (12.)	48.1 (11.0)	46.6 (11.7)	48.1 (11.0)	49.1 (9.0)	0.836	0.25	46.9 (11.9)	44.4 (13.3)	0.373	−0.16
Physical function	66.8 (20.8)	73.1 (18.5)	78.3 (17.9)	79.5 (15.2)	78.6 (17.0)	<0.001	0.61	75.2 (21.1)	76.4 (20.0)	0.013	0.46
Social function	72.1 (23.3)	80.8 (18.2)	78.7 (19.8)	84.1 (18.5)	81.0 (18.8)	0.020	0.38	76.8 (22.2)	75.7 (25.0)	0.543	0.15
RL physical	59.1 (40.7)	75.0 (36.2)	73.8 (36.7)	73.8 (33.0)	68.5 (41.7)	0.062	0.36	64.9 (42.8)	62.1 (42.2)	0.737	0.07
RL Emotional	70.5 (40.8)	75.2 (37.2)	75.8 (37.7)	81.7 (32.3)	81.7 (32.3)	0.074	0.35	77.0 (40.0)	64.8 (41.2)	0.535	−0.14
Mental health	69.0 (17.3)	76.1 (15.7)	72.4 18.4)	75.6 (16.8)	75.8 (16.7)	0.016	0.38	71.8 (17.2)	70.2 (19.9)	0.853	0.07
Vitality	51.5 (18.7)	57.3 (21.1)	58.3 (22.2)	61.5 (18.5)	60.5 (18.2)	0.002	0.53	56.4 (23.1)	54.3 (20.8)	0.704	0.15
Pain	70.8 (20.8)	77.9 (15.2)	76.7 (19.1)	74.6 (18.0)	73.7 (20.4)	0.236	0.18	72.9 (20.8)	73.5 (19.5)	0.466	0.13
General health	49.0 (17.7)	59.4 (18.8)	62.6 (17.9)	64.4 (17.8)	61.1 (18.9)	<0.001	0.68	58.3 (21.4)	57.0 (20.0)	0.006	0.45
Health transition	47.2 (24.2)	66.3 (21.7)	72.6 (22.2)	72.0 (21.1)	75.0 (19.1)	<0.001	1.15	64.0 (23.7)	52.9 (25.6)	0.515	0.24

Abbreviations: PCS, Physical Component Summary score; MCS, Mental Component Summary score. ES, effect size; RL, role limitation. Values shown are means (standard deviations). *^¥^* Outcomes between baseline and 1 year and between baseline and 2 years were compared with paired sample *t*-tests.

**Table 3:** Secondary outcomes: physical outcomes, exercise capacity, lipid profile, and HbA1c during the first 2 years of the IBO (*n* = 44).

	Base- Line	3 Months	6 Months	9 Months	1 Year	*p ^¥^*	ES	1.5 Years	2 Years	*p ^¥^*	ES
Weight (kg)	114.0 (16.6)	110.1 (16.1)	109.1 (16.6)	108.3 (16.5)	106.6 (14.9)	<0.001	−0.45	109.4 (16.5)	110.5 (18.1)	0.003	−0.21
Body mass index (kg/m^2^)	38.5 (4.7)	37.0 (4.8)	36.7 (5.0)	36.3 (4.7)	36.0 (4.4)	<0.001	−0.53	36.7 (5.0)	37.3 (5.3)	0.003	−0.26
Waist circumference (cm)	121.7 (12.3)	117.2 (13.3)	116.3 (13.8)	114.8 (12.3)	114.5 (12.3)	<0.001	−0.59	115.3 (13.7)	116.5 (14.3)	<0.001	−0.42
Hip circumference (cm)	130.6 (9.2)	125.2 (10.0)	126.0 (9.6)	125.1 (9.0)	125.0 (8.5)	<0.001	−0.61	124.2 (10.8)	125.5 (9.5)	<0.001	−0.55
Body fat (%)	46.7 (5.2)	44.5 (5.6)	44.3 (6.5)	43.3 (5.9)	43.6 (6.3)	<0.001	−0.60	44.0 (6.0)	44.8 (5.8)	0.004	−0.37
Visceral fat (%)	15.7 (5.9)	14.3 (5.3)	14.1 (4.9)	14.4 (5.5)	14.1 (5.0)	<0.001	−0.27	14.9 (6.0)	15.1 (6.0)	0.179	−0.10
VO_2_-max (ml/kg/min)	20.7 (4.8)		24.2 (5.5)		24.7 (5.6)	0.030	0.38	25.2 (5.5)	25.3 (5.6)	<0.001	0.96

Abbreviations: ES, effect size. Values shown are means (standard deviations). *^¥^* Outcomes between baseline and 12- and between baseline and 2 years were compared with paired sample *t*-tests.

**Table 4:** Secondary outcomes: Lipid profile and HbA1c during the first 2 years of the IBO (*n* = 44).

	Baseline ^#^	3 Months	6 Months	9 Months	1 Year	*p ^¥^*	ES	1.5 Years	2 Years	*p ^¥^*	ES
TC (mmol/L)	4.8 (0.8)	4.5 (7.3)	4.5 (0.8)	4.6 (0.9)	5.0 (0.8)	0.549	0.00	5.0 (0.7)	5.1 (0.9)	0.295	0.38
HDL-c (mmol/L)	1.3 (0.3)	1.2 (0.2)	1.2 (0.3)	1.3 (0.3)	1.3 (0.3)	0.138	0.00	1.3 (0.3)	1.3 (0.3)	0.363	0.00
TC/HDL-c	3.9 (1.1)	4.0 (1.1)	3.8 (1.2)	3.8 (1.2)	3.9 (1.4)	0.173	0.17	4.1 (1.2)	4.2 (1.3)	0.718	0.27
LDL-c (mmol/L)	2.9 (0.8)	2.7 (0.7)	2.5 (0.8)	2.7 (0.8)	3.0 (0.8)	0.292	0.13	3.0 (0.8)	3.2 (0.8)	0.935	−0.38
TG (mmol/L)	1.6 (1.3)	1.4 (0.7)	1.5 (0.7)	1.4 (0.7)	1.4 (0.8)	0.440	0.00	1.6 (0.8)	1.5 (0.8)	0.287	0.08
HbA1c (mmol/mol)	41.0 (9.6)	39.2 (7.2)	38.5 (6.5)	39.3 (6.3)	40.5 (10.9)	0.975	0.01	39.7 (6.6)	41.4 (8.1)	0.699	−0.04

Abbreviations: ES, effect size; TC, total cholesterol level; HDL-c, high-density lipoprotein level; LDL-c, low-density lipoprotein level; TG, triglycerides level. Values shown are means (standard deviations). ^#^ Values of all participants who were still participating at 2 years (*n* = 44). *^¥^* Outcomes between baseline and 12- and between baseline and 2 years were compared with paired sample *t*-tests.

**Table 5:** Secondary outcomes: RSES, CIS, SCL-90, and UCL scores during the first 2 years of the IBO (*n* = 44).

	Base- Line ^#^	3 Months	6 Months	9 Months	1 Year	*p ^¥^*	ES	1.5 Years	2 Years	*p ^¥^*	ES
RSES	20.5 (5.0)		21.4 (5.6)		22.3 (4.9)	0.008	−0.14		20.9 (5.4)	0.765	−0.05
CIS–Total score	77.0 (24.1)		67.4 (26.8)		66.2 (22.6)	0.001	0.56		70.4 (26.0)	0.900	0.30
Fatigue	38.3 (11.3)		30.2 (12.8)		29.7 (11.5)	<0.001	0.76		31.4 (11.3)	0.001	0.64
Concentration	15.6 (8.7)		15.3 (7.7)		16.2 (7.9)	0.330	0.15		16.1 (8.0)	0.722	0.06
Motivation	12.8 (5.0)		11.8 (4.6)		11.8 (4.6)	0.083	0.27		12.8 (5.3)	1.000	0.00
Physical activity	10.4 (5.2)		10.2 (5.0)		9.1 (4.6)	0.082	0.28		10.5 (5.2)	0.875	0.03
SCL-90											
Anxiety	13.2 (3.0)	13.2 (3.0)	12.6 (3.3)	12.3 (2.8)	12.0 (2.3)	0.005	−0.50	13.6 (6.4)	13.4 (3.7)	0.811	0.05
Agoraphobia	8.3 (1.8)	7.9 (1.7)	7.7 (1.2)	7.7 (1.5)	7.7 (1.5)	0.079	−0.30	9.2 (9.3)	8.1 (1.8)	0.232	0.23
Depression	26.9 (8.7)	23.5 (6.8)	23.9 (7.8)	22.7 (6.2)	21.7 (5.9)	<0.001	−0.69	22.1 (8.0)	26.2 (10.0)	0.745	0.06
Somatization	19.1 (5.5)	18.3 (4.5)	17.4 (4.0)	17.4 (3.5)	16.9 (3.4)	0.015	−0.43	19.8 (14.4)	17.8 (3.8)	0.106	0.31
Insufficiency of thinking and acting	16.3 (5.9)	14.5 (4.2)	13.9 (4.1)	13.7 (4.1)	13.6 (3.6)	0.079	−0.65	15.0 (9.4)	14.9 (4.8)	0.122	0.30
Interpersonal sensitivity	28.1 (10.3)	24.8 (6.4)	25.0 (5.5)	23.5 (4.9)	22.8 (3.7)	0.002	−0.57	22.8 (5.1)	25.8 (7.6)	0.019	0.46
Hostility	7.8 (2.0)	7.3 (1.4)	7.4 (1.3)	6.9 (1.1)	7.2 (1.1)	0.061	−0.30	7.8 (4.5)	7.4 (1.7)	0.047	0.39
Sleep difficulties	6.1 (2.5)	6.1 (3.0)	5.8 (2.8)	6.2 (2.7)	5.8 (2.5)	0.294	−0.18	6.6 (7.1)	5.8 (2.5)	0.102	0.32
UCL											
Active confronting	19.2 (4.3)		19.1 (4.1)		19.1 (4.1)	0.877	−0.00		18.2 (3.9)	0.087	0.31
Palliative reaction	17.9 (3.7)		16.6 (3.3)		17.7 (3.7)	1.000	−0.00		18.0 (3.7)	0.828	0.04
Avoidance	16.9 (3.3)		15.7 (4.2)		15.7 (3.0)	0.001	−0.62		15.7 (3.6)	0.011	0.48
Seeking social support	13.5 (4.1)		14.3 (4.1)		14.2 (4.2)	0.082	−0.29		13.9 (4.0)	0.551	−0.11
Passive reaction pattern	12.0 (3.4)		11.2 (2.9)		10.8 (2.5)	0.002	−0.54		11.4 (3.1)	0.143	0.27
Expressing emotions	6.4 (2.0)		6.0 (1.3)		6.0 (1.4)	0.183	−0.22		6.0 (1.7)	0.057	0.34
Reassuring thoughts	11.6 (2.6)		12.1 (2.6)		11.5 (3.1)	0.935	−0.01		11.2 (2.9)	0.325	0.18

Abbreviations: ES, effect size; RSES, Rosenborg Self-Esteem Scale; CIS, Checklist Individual Strength; SCL-90, Symptom Checklist-90; UCL, Utrecht Coping List. Values shown are means (standard deviations);. ^#^ Values of all participants who were still participating at 2 years. *^¥^* Outcomes between baseline and 12- and between baseline and 2 years were compared with paired sample *t*-tests.

## Data Availability

The datasets presented in this article are not readily available because the data are part of an ongoing study.
